# Systematic Metabolic Engineering and Model‐Guided Optimization for High‐Level Production of L‐Theanine from Xylose in *Escherichia coli*

**DOI:** 10.1002/advs.202521440

**Published:** 2026-01-21

**Authors:** Haolin Han, Boyuan Xue, Guangqi Shan, Meng Meng, Shaojie Wang, Haijia Su

**Affiliations:** ^1^ State Key Laboratory of Green Biomanufacturing National Energy R&D Center for Biorefinery Beijing Key Laboratory of Green Chemicals Biomanufacturing Beijing Synthetic Bio‐Manufacturing Technology Innovation Center Beijing University of Chemical Technology Beijing P. R. China

**Keywords:** D‐xylose, *Escherichia coli*, L‐theanine, weimberg pathway, γ‐Glutamylmethylamide synthetase

## Abstract

Lignocellulosic biomass represents a promising sustainable feedstock for biomanufacturing, yet the efficient conversion of its dominant pentose, D‐xylose, into high‐value α‐ketoglutarate derivatives like L‐theanine remains challenging due to the inherent carbon loss and low yield of conventional metabolic pathways. To overcome this limitation, a novel microbial platform was developed by reconstituting the carbon‐conserving Weimberg pathway in *E. coli*, enabling the direct and *de novo* biosynthesis of L‐theanine from xylose in just 7 enzymatic steps. Through comprehensive metabolic engineering, including the blocking of competitive pathways, enhancing the precursor supply, and fine‐tuning cofactor balance, the overproducing strain TH 4‐4 achieved a titer of 9.94 g/L and a yield of 0.33 g/g. Furthermore, flux balance analysis of enzyme‐constrained metabolic network model was used to quantitatively assess metabolic trade‐offs, and a two‐stage microaerobic‐aerobic cultivation strategy was implemented, resulting in the highest titer of 14.31 g/L and a yield of 0.48 g/g, representing a 2811.4‐fold increase compared to the original strain. Finally, a fed‐batch fermentation of the engineered strain achieved a titer of 95.42 g/L, a yield of 0.55 g/g xylose, and a productivity of 1.33 g/L/h. This work pioneers the high‐level production of L‐theanine from xylose and provides a transformative framework for the sustainable valorization of lignocellulosic sugars into valuable TCA cycle derivatives.

## Graphical abstract

This study is pioneering in constructing the shortest known synthetic pathway for L‐theanine production from xylose within *E coli*. Through comprehensive metabolic engineering strategies, our engineered strain achieved the highest reported L‐theanine titer from xylose, with a titer of 95.42 g/L, and a yield of 0.55 g/g. This work establishes a scalable and economically attractive platform for the sustainable production of L‐theanine as well as other TCA‐derived biochemicals.

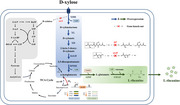

## 1 Introduction

Lignocellulose represents the most abundant biomass resource in nature, constituting over half of the total biomass. Approximately 15–35% of lignocellulose consists of heteropolysaccharides, known as hemicellulose, which are primarily composed of glucose and xylose. Therefore, xylose resources are abundant in nature, and the development of microbial cell factories capable of efficiently metabolizing xylose is of great importance for the complete utilization of lignocellulose [1, 2, 3]. Traditionally, the utilization of xylose has been limited to its reduction and hydrogenation into xylitol, a non‐caloric sweetener, and food antioxidants, among other uses. However, the demand for products in these areas is relatively small, unable to drive the extensive development of the vast xylose resources. Compared to glucose, most microorganisms metabolize xylose less efficiently, which further restricts its broader application [4, 5].

In conventional microbial cell factories [6, 7] (e.g., *Saccharomyces cerevisiae* and *Escherichia coli*), D‐xylose metabolism primarily occurs through the pentose phosphate pathway (PPP), where it is converted to pyruvate and subsequently oxidized to acetyl‐CoA for entry into the TCA cycle [8]. However, this process suffers from inherent carbon loss, with 33% of substrate carbon being wasted as CO_2_ during Acetyl‐CoA formation. Additionally, the multi‐step nature of these traditional metabolic pathways imposes limitations on both product yield and production rate [9, 10].

Recent studies have highlighted an alternative, non‐phosphorylated pentose conversion pathway found in some microorganisms, known as the Weimberg pathway [11]. This pathway begins with the dehydrogenation of D‐xylose to D‐xylonolactone catalyzed by xylose dehydrogenase, followed by a hydrolysis and ring‐opening reaction catalyzed by xylonolactonase to form D‐xylonate. Then, 2‐keto‐3‐deoxy‐D‐xylonate dehydratasecatalyzes the dehydration to produce 2‐keto‐3‐deoxy‐D‐xylonate (D‐KDX). Subsequently, D‐KDX dehydratase catalyzes the formation of 2,5‐dioxopentanoate, which is then oxidized to α‐ketoglutarate by 2,5‐dioxopentanoate dehydrogenase. This process directly converts the C5 skeleton into a TCA cycle intermediate in just 5 steps without the production of CO_2_ [10, 12, 13]. The Weimberg pathway, as an efficient and environmentally friendly pentose assimilation pathway, shows significant potential for replacing conventional pentose and hexose metabolic pathways.

L‐Theanine, a unique amino acid predominantly found in tea plants, constitutes about 60%–70% of the free amino acids in tea leaves, accounting for 1%–2% of the dry weight of the leaves [14]. It was first isolated by Japanese scholars from Gyokuro green tea, with the chemical name N‐ethyl‐γ‐glutamine [15]. L‐Theanine imparts a fresh taste and unique flavor to tea, and its content in tea leaves can, to some extent, affect the quality and price of the tea. Beyond its distinctive flavor, L‐theanine has numerous beneficial effects on human health, such as alleviating stress, improving sleep quality, regulating neurotransmitter transmission, inhibiting hypertension, antioxidant and antitumor properties, as well as enhancing memory [16, 17]. The expanding applications of L‐theanine have driven substantial industrial investment to meet growing market demands.

Given these advantages, microbial fermentation has emerged as the predominant strategy for L‐theanine production. Significant progress has been made through metabolic engineering in various microbial hosts. For instance, heterologous expression of a maize‐derived γ‐glutamylmethylamide synthetase (GMAS) in *Corynebacterium glutamicum* GDK‐9 enabled the production of 42 g/L L‐theanine [18]. Yang et al., [19] employed computer‐aided design to engineer GMAS_Pa_ from *Paracoccus aminophilus*, and combined this with metabolic flux optimization to achieve a titer of 44.12 g/L in *C. glutamicum*—the highest reported in this host to date. Fan et al., [20] expressed a GMAS in *E. coli* using an inducible T7 expression system, reaching a high titer of 70.6 g/L from glucose in a 5 L fermenter.

While efficient production from glucose has been established, the valorization of xylose remains a critical goal for fully leveraging lignocellulosic biomass. A previous attempt to produce L‐theanine from xylose was made by engineering *Pseudomonas putida* with the Weimberg pathway [21]. However, the final titer of 4 g/L L‐theanine in fed‐batch fermentation indicated substantial challenges in pathway efficiency and scalability, suggesting that the potential of xylose as a feedstock for high‐level production had not yet been fully realized.

**FIGURE 1:**
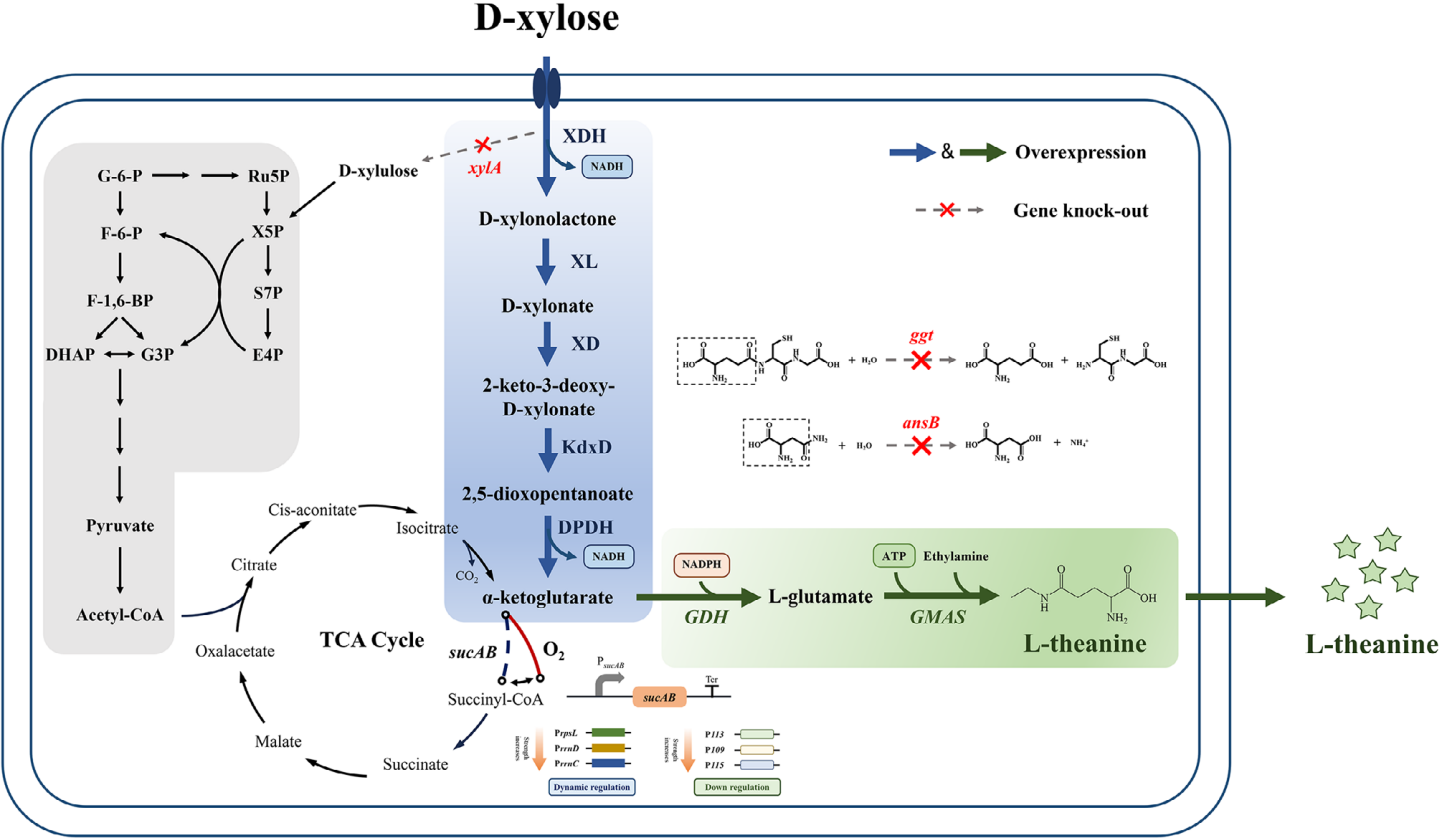
Metabolic network modification and optimization strategies for L‐theanine production in *Escherichia coli*. The blue background represents the D‐xylose metabolism module, where xylose is converted to D‐xylonolactone by D‐xylose dehydrogenase (XDH), followed by hydrolysis and ring‐opening reactions catalyzed by D‐xylonolactonase (XL) to form D‐xylonate. Then, D‐xylonate dehydratase (XD) catalyzes dehydration to produce 2‐keto‐3‐deoxy‐D‐xylonate (D‐KDX). Subsequently, D‐KDX dehydratase (KdxD) catalyzes the formation of 2,5‐dioxopentanoate, which is finally oxidized to α‐KG by 2,5‐dioxopentanoate dehydrogenase (DPDH). The green background represents the L‐theanine synthesis module, where α‐KG is ultimately converted to L‐theanine through the action of glutamate dehydrogenase (GDH) and γ‐glutamylmethylamide synthetase (GMAS).

In this study, we demonstrate for the first time the efficient biosynthesis of L‐theanine from xylose in *E. coli* using a systematic engineering and model‐guided optimization strategy (Figure 1). By implementing comprehensive metabolic engineering approaches, including blocking competitive and degradation pathways, optimizing precursor supply, and fine‐tuning central metabolic flux, the engineered strain achieved a titer of 14.3 g/L in shake flasks and a remarkable 95.42 g/L in a 5 L bioreactor, representing the highest reported titer for *de novo* L‐theanine biosynthesis. This work establishes a scalable and economically attractive platform for the sustainable production of L‐theanine as well as other TCA‐derived biochemicals.

## 2 Materials and Methods

### 2.1 Microorganisms, Plasmids, and Modification of Chromosome Genes

The microorganisms and plasmids used in this study are listed in Table S1. Specifically, *E. coli* JM109 was employed as a host for plasmid construction, and *E. coli* BL21(DE3) was used as the host for strain engineering and L‐theanine production experiments. The pET Duet and pACYC Duet plasmids were employed as expression vectors, and the CRISPR‐Cas9 system based on pEcCas9 and pEc‐gRNA plasmids was applied for efficient genome editing of the engineered *E. coli*.

Gene integration into the *E. coli* BL21(DE3) genome was performed using the CRISPR/Cas9 system. First, the homologous arms of the target gene and the pEc‐gRNA vector plasmid were amplified by PCR and fused to construct the donor DNA. The donor DNA, together with the target‐specific gRNA, was then introduced into host cells already containing the pEcCas9 plasmid. Transformants were selected by culturing on LB agar plates supplemented with 50 mg/L chloramphenicol and kanamycin at 37°C for 12–16 h. Subsequently, single colonies were preliminarily screened by colony PCR, and randomly selected positive clones were further verified by DNA sequencing. After correctly edited colonies were obtained, the donor DNA plasmid was eliminated by sequential growth in LB medium containing 0.2% rhamnose, followed by removal of the pEcCas9 plasmid in LB medium supplemented with 1% sucrose.

### 2.2 Heterologous Expression of L‐Theanine Synthesis Pathway

All primers used for strain construction are listed in Table S2. The genes employed in this study and their sources are as follows: the *xylB*, *xylC*, *xylA*, and *xylX* genes from *C. crescentus*, the GMAS‐A gene from *P. aminovorans* JCM7685, the GMAS‐S gene from *P. syringae*, as well as the GDH‐1 gene from *Amphibacillus xylanus* and the GDH‐2 gene from *Clostridium difficile*, which were all chemically synthesized by Genewiz (Suzhou, China) following codon optimization. Additionally, the *yjhG* gene was directly amplified by PCR from the *E. coli* MG1655 genome, and the GDH‐3 and GDH‐4 genes were amplified from *E. coli* and *C. glutamicum*, respectively.

Subsequently, the *xylB*, *xylC*, *yjhG*, *xylA*, and *xylX* gene fragments were collectively cloned into the pACYC‐Duet plasmid using Gibson Assembly. Meanwhile, the respective GMAS genes were paired with their corresponding GDH genes and cloned into the pET‐Duet plasmid. Finally, all recombinant plasmids were individually transformed into *E. coli* BL21(DE3) competent cells via electroporation.

### 2.3 Production of L‐Theanine by Fermentation in Shake Flasks

The preserved engineered strain was first inoculated into a test tube containing 5 mL of seed medium and cultured at 37°C with shaking at 200 rpm for 12 h. Then, 1 mL of the culture was transferred into a 250 mL Erlenmeyer flask containing 30 mL of seed medium and incubated for another 12 h to prepare the seed culture. Finally, the seed culture was inoculated at 1% (v/v) into a 250 mL flask containing 26 mL of fermentation medium. At the beginning of fermentation, 1 mM IPTG was added in a single dose to induce the expression of the full‐pathway proteins. Fermentation was carried out at 37°C with shaking at 200 rpm for a total duration of 60 h. The pH of the fermentation broth was monitored using a Mettler micro‐pH meter and maintained at a constant level by dropwise addition of NaOH solution. In addition, 2 g/L of a 50% (m/v) ethylamine hydrochloride solution was supplemented every 12 h.

The seed medium (per liter) contained 5 g yeast extract, 10 g tryptone, and 10 g NaCl. The fermentation medium consisted of M9 minimal medium supplemented with the appropriate antibiotic, and added with (per liter) 30 g xylose, 5 g yeast extract, 75 mM MOPS (pH 7.4), 2 mM MgSO_4_, 10 µM FeSO_4_, 0.1 mM CaCl_2_, and trace elements.

### 2.4 Production of L‐Theanine in a 5‐L Bioreactor

A single colony was first inoculated into a test tube containing 10 mL of LB medium and cultured at 37°C with shaking at 200 rpm for 12 h. The culture was then transferred into a 500 mL Erlenmeyer flask containing 100 mL of LB medium and incubated under the same conditions for another 12 h. Subsequently, the culture was inoculated into a 5 L bioreactor (Mancang, Beijing, China) containing 2 L of fresh amplification medium. The cultivation conditions were set as follows: temperature 37°C, pH controlled by automatic feeding of ammonium hydroxide (25%, v/v), and dissolved oxygen (DO) maintained above 20% by adjusting the agitation speed and aeration rate. When the OD_600_ of the culture reached 12–15, 500 mL was retained as the seed culture, and 2 L of fresh amplification medium was immediately supplemented into the reactor to initiate the fermentation stage. The fermentation conditions (temperature, pH, and DO) were kept the same as those in the seed culture stage. After the initial xylose was depleted, a fed‐batch process was started with a 500 g/L xylose solution to maintain the residual sugar concentration below 5 g/L. Simultaneously, a 50% (m/v) ethylamine hydrochloride solution was automatically fed. If foaming occurred, a silicone‐based antifoam agent was added dropwise. Throughout the fermentation process, samples were taken every 4 h to measure OD_600_ and analyze metabolite concentrations.

The amplification medium was based on M9 minimal medium supplemented with appropriate antibiotics and contained the following components per liter: 20 g xylose, 5 g yeast extract, 5 g peptone, 10 g glucose, 75 mM MOPS (pH 7.4), 2 mM MgSO_4_, 20 µM FeSO_4_, 0.1 mM CaCl_2_, and trace elements.

### 2.5 Metabolic Flux Analysis Method

This study employs a pathway analysis approach with genome‐scale metabolic models for the rational design of microbial metabolic pathways. We utilized the extensively adopted *E. coli* GEM: iML1515 for screening, which is optimized for constructing heterologous pathways through its integration of rich biological databases like BioCyc, EcoCyc, and KEGG. To enhance the prediction accuracy, enzyme kinetic constraints were incorporated into the GEM, leading to model reconstruction and the embedding of new constraints (Equation 3).

(1) S∗v=0

(2) $$v_{min}\leq v\leq v_{max}$$

(3) $$\sum_{i=1}^{n}\frac{v_{i}\cdot MW_{i}}{k_{\mathrm{cat}}}\leq ENZ_{E.coli}$$

*S* represents the stoichiometric matrix of the GEM, and *v* represents the flux vector of all reactions, since all reversible reactions were disassembled into irreversible reactions, the lower limit of the reaction fluxes was *v_min_* =  0.

To analyze the differences in xylose metabolism among the Weimberg pathway, Dahms pathway, and PP pathway, maximum growth and substrate uptake gradients were set based on reaction flux bounds under enzyme constraints (Equation 2), in order to evaluate the theoretical capacity of L‐theanine synthesis from xylose via these different pathways.

Furthermore, to provide additional guidance for optimizing L‐theanine synthesis conditions, we imposed additional conditions on the enzyme‐constrained GEM with the objectives of: (i) maximum biomass production, (ii) maximum L‐theanine production under basal growth conditions, and (iii) minimal growth‐associated metabolism. The flux differences of key reactions and pathways in the steady‐state flux balance analysis results were examined, and complete metabolic pathways were mapped with flux distributions to achieve visualization.

### 2.6 Analytical Methods

Cell growth was monitored by measuring the absorbance at 600 nm (OD600). L‐theanine, L‐glutamic acid, α‐ketoglutarate, and xylose were detected using high‐performance liquid chromatography (HPLC; Agilent Technologies, USA).

Fermentation broth samples were centrifuged at 12 000 rpm for 2 min, and the resulting supernatant was collected for medium component analysis. For the detection of L‐theanine and L‐glutamic acid, samples were first derivatized with DNFB (2,4‐dinitrofluorobenzene). Separation was then performed on a Waters Symmetry C18 column (250 × 4.6 mm, 5 µm) maintained at 30°C, using a gradient elution program. Mobile phase A consisted of 50 mM ammonium acetate, and mobile phase B was 50% acetonitrile. The gradient was set as follows: mobile phase B increased from 0% to 100% over 35 min. The flow rate was 0.5 mL/min. α‐Ketoglutarate and xylose were analyzed using an organic acid column with 5 mM H_2_SO_4_ as the mobile phase, at a flow rate of 0.6 mL/min.

## 3 Results

### 3.1 Comparative Pathway Analysis of Xylose Metabolism Pathways

**FIGURE 2:**
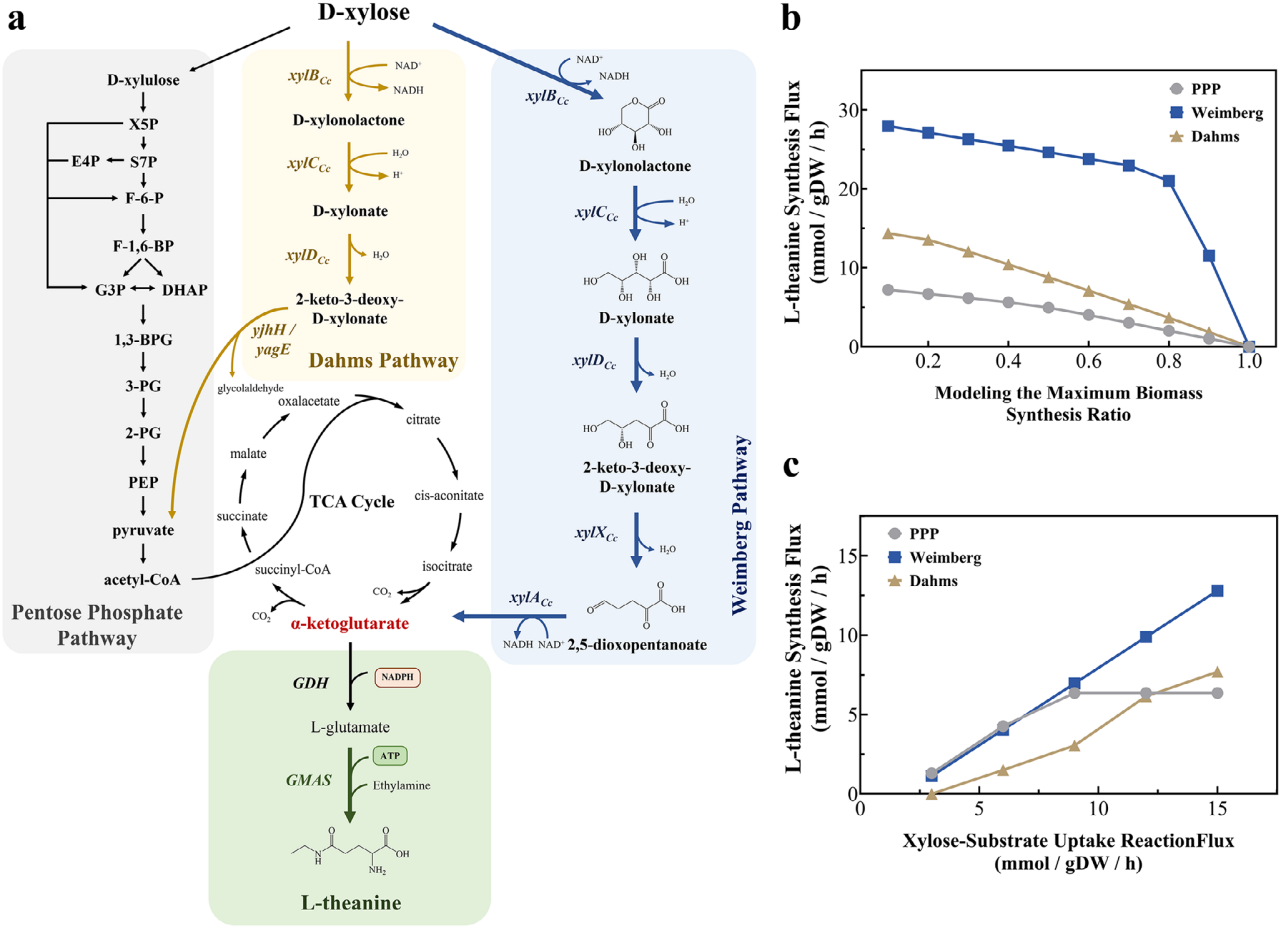
Comparative pathway analysis of xylose metabolism pathways. (a) Schematic diagram of xylose metabolic pathway. Xylose assimilated routes are indicated in different colors. The gray pathway is Pentose Phosphate pathway, the yellow pathway is Dahms pathway, and the blue pathway is the Weimberg pathway. (b) The maximum biomass synthesis ratio of the Weimberg pathway, Dahms pathway, and PPP was predicted by metabolic flux analysis. (c) The xylose‐substrate uptake reactionflux of the Weimberg pathway, Dahms pathway, and PP pathway was predicted by metabolic flux analysis. Enzymes encoded by the genes shown are *xylBCc*, xylose dehydrogenase; *xylCCc*, xylonolactonase; *xylDCc*, xylonate dehydratase; *xylXCc*, d‐KDX dehydratase; *xylACc*, 2,5‐dioxopentanoate dehydrogenase.

The biosynthesis of high‐value chemicals from xylose represents a highly promising strategy for sustainable bioproduction due to its economic viability and broad industrial applications. However, conventional xylose utilization in *Escherichia coli* through the pentose phosphate pathway (PPP) faces critical limitations. For instance, the *de novo* synthesis of L‐theanine via PPP involves a complex enzymatic cascade of approximately 17 steps (Figure 2a, gray), which imposes significant thermodynamic constraints. Moreover, CO_2_ loss during the irreversible decarboxylation of pyruvate to Acetyl‐CoA limits the product yield to no more than 0.83 mol/mol xylose (Table 1).

**TABLE 1:** Comparison of the xylose nonphosphorylation pathway and phosphorylation pathway.

	Weimberg pathway	Dahms pathway	PP pathway
Initial substrate	Xylose	Xylose	Xylose
Product	L‐Theanine	L‐Theanine	L‐Theanine
Yield	100％	50％	83％
Number of reaction steps	7	11	17
Energy (ATP)	−1.00	−1.50	+0.16
Reducing power	+2 NADH	+1 NADPH	+1 NADH
	−1 NADPH		
Coenzyme required	—	CoA‐SH	CoA‐SH

To circumvent these thermodynamic and carbon conservation challenges, non‐phosphorylated metabolic routes have garnered increasing attention. Both the Dahms and Weimberg pathways initiate with the enzymatic oxidation of D‐xylose to D‐xylose lactone through xylose dehydrogenase, followed by lactonase‐mediated hydrolysis to yield D‐xylonate. Subsequent dehydration via 2‐keto‐3‐xylose dehydratase generates 2‐keto‐3‐deoxy‐D‐xylonate (KDX) as a key intermediate. Diverging at this metabolic node, the Dahms pathway employs an aldolase to cleave KDX into pyruvate and acetaldehyde, ultimately requiring 11 catalytic steps for L‐theanine synthesis (Figure 2a, yellow). Despite its reduced reaction complexity compared to PPP, this pathway still suffers from carbon loss through CO_2_ emission during intermediate processing, limiting the theoretical yield to only 0.5 mol/mol xylose and thus significantly restricting its practical application [10, 12, 13].

The Weimberg pathway achieves complete carbon conservation from xylose to L‐theanine in just 7 steps (Figure 2a, blue). This optimized route eliminates decarboxylation‐mediated carbon loss by directly channeling xylose‐derived metabolites into the TCA cycle, enabling stoichiometric conversion of 1 mole xylose to 1 mole L‐theanine. Such carbon‐efficient transformation, coupled with reduced enzymatic complexity and energy expenditure, establishes the Weimberg pathway as a superior framework for biosynthesis of TCA cycle‐derived compounds from xylose.

To further investigate the metabolic efficiency of these pathways, we employed a genome‐scale metabolic network model (GEM) to simulate and compare flux distributions between the PPP, Dahms pathway, and Weimberg pathway under varying substrate consumption and biomass constraints. Simulations revealed that the Weimberg pathway consistently exhibited the highest flux for L‐theanine synthesis, outperforming both the PPP and the Dahms pathway (Figure 2b,c). Consequently, the Weimberg pathway boasts a higher theoretical conversion rate, enabling the accumulation of higher concentrations of L‐theanine synthesis precursors, thereby being more conducive to L‐theanine production.

### 3.2 Engineering the Weimberg Pathway for Enhanced Xylose‐to‐α‐KG Conversion

To establish the Weimberg pathway in *E. coli*, we first deleted the native *xylA* gene, which encodes xylose isomerase in the PPP, to block competing xylose metabolism. Next, we heterologously expressed a codon‐optimized operon containing the *xylBCDXA* genes from *C. crescentus*, generating the engineered strain W1. To validate the functional replacement of PPP with the Weimberg pathway, we performed growth assays using xylose as the sole carbon source.

**FIGURE 3:**
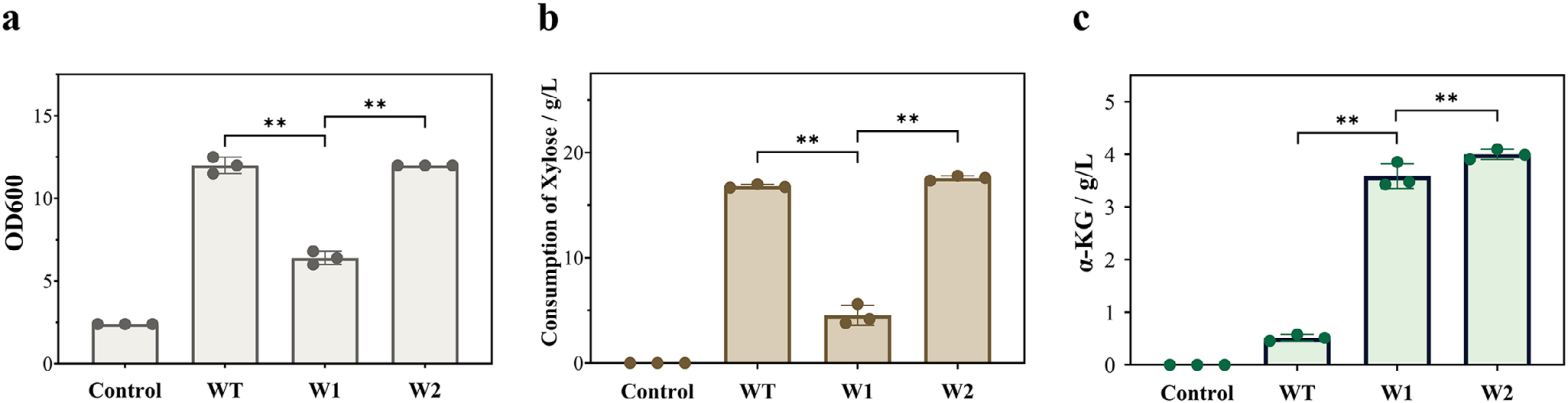
Construction and optimization of the Weimberg pathway. Comparison of (a) biomass formation, (b) xylose consumption, (c) α‐KG production. ^*^ *p* < 0.05 and ^**^ *p* < 0.01 indicate the significance level between the two engineered strains. Each experiment was repeated three times, and data are represented as the means of three replicates, and bars represent the standard deviations.

As shown in Figure 3a, the wild‐type *E. coli* BL21 (retaining PPP activity) exhibited robust growth, while the Δ*xylA* mutant failed to utilize xylose, confirming the essential role of *xylA* in native xylose metabolism. However, although strain W1 was capable of growing on xylose via the Weimberg pathway, its growth rate was significantly lower than that of the wild‐type strain. Previous studies indicated that the activity of xylonate dehydratase, encoded by *xylD*, may be the rate‐limiting step in the conversion of xylose to α‐ketoglutarate (α‐KG) [12]. Notably, *E. coli* K‐series strains (e.g., MG1655) naturally possess *yjhG* and *yagF* encoding putative xylonate dehydratases, with *yjhG* gene serving as the primary enzyme responsible for converting xylonate to KDX.

To address this limitation, we replaced the *C. crescentus* derived *xylD* gene with the *yjhG* gene from *E. coli* MG1655, generating strain W2. Growth capability was fully restored in ‌strain W2, with markedly enhanced xylose utilization efficiency (Figure 3b), demonstrating the successful reconstitution of the pathway. Notably, strain W2 accumulated 4.00 ± 0.10 g/L α‐KG, representing an 8.0‐fold increase over the wild‐type strain (0.52 ± 0.06 g/L) (Figure 3c). Collectively, these modifications establish a robust chassis strain that efficiently channels xylose into the TCA cycle intermediate α‐KG.

### 3.3 *De Novo* Synthesis of L‐Theanine from Xylose

**FIGURE 4:**
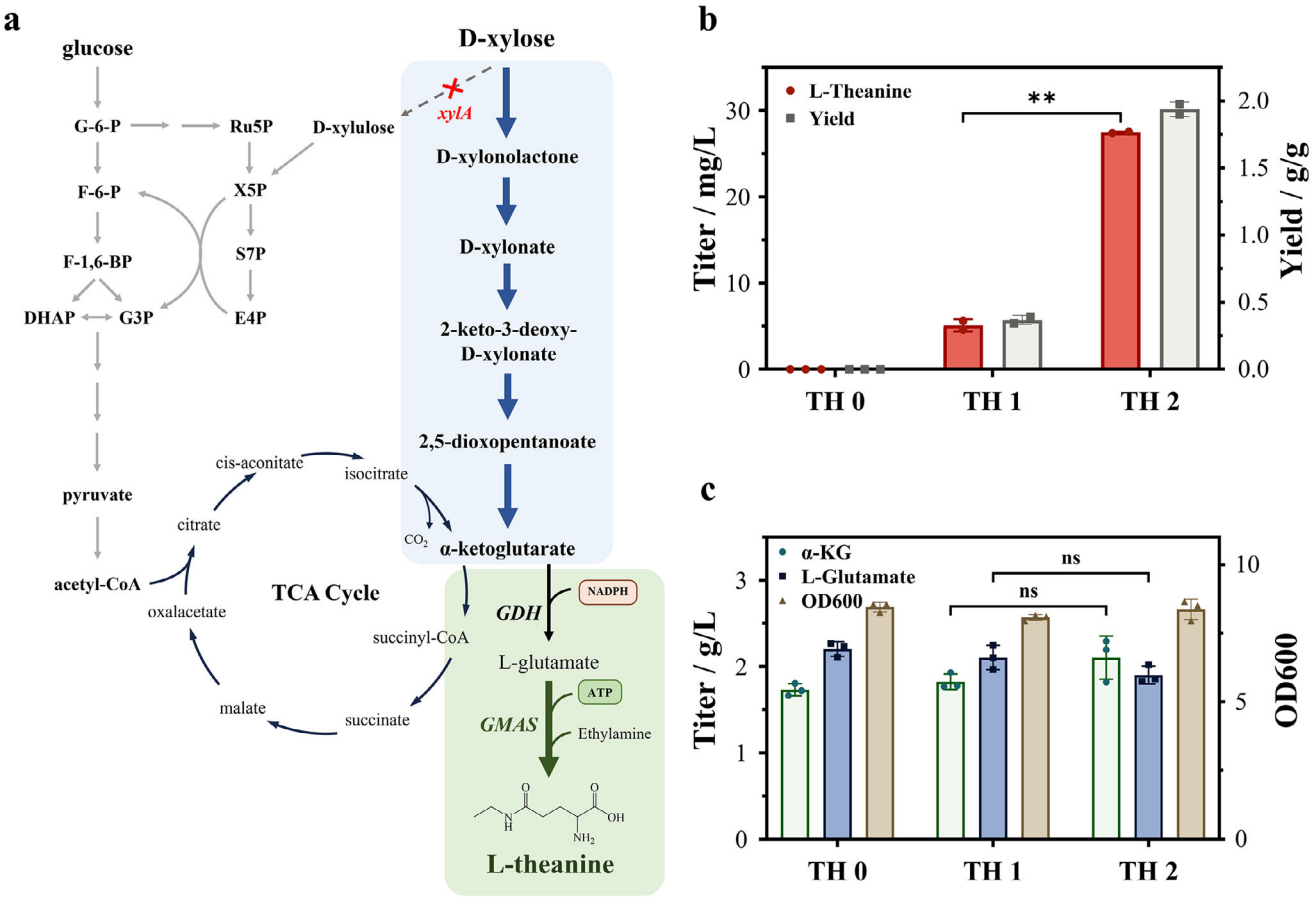
*De novo* synthesis of L‐theanine from xylose. (a) Schematic diagram of L‐theanine synthesis pathway. Comparison of (b) L‐theanine production and yield, (c) glutamate, α‐KG accumulation, and biomass formation between strains without overexpression of GMAS (TH 0), strains overexpressing GMASPs (TH 1), and strains overexpressing GMAS‐A (TH 2).

Having established the enhanced α‐KG production chassis, we next sought to channel this TCA cycle intermediate into L‐theanine synthesis via glutamine‐dependent transamination (Figure 4a). The γ‐glutamylmethylamide synthetase (GMAS), originally characterized in methylotrophic organisms for methylamine metabolism, was selected for its broad substrate promiscuity toward alkylamines. Two phylogenetically distinct GMAS enzymes, GMAS‐A [19, 20] from *Paracoccus aminovorans* JCM7685 and GMAS‐S [22] from *Pseudomonas syringae*, were codon‐optimized and introduced into the xylose‐utilizing W2 chassis, generating strains TH 1 (GMAS‐A) and TH 2 (GMAS‐S), with empty vector control TH 0.

As shown in Figure 4b, both engineered strains were capable of synthesizing L‐theanine. However, strain TH 2 exhibited a production capacity 5.4‐fold higher than that of strain TH 1 (5.09 ± 0.71 mg/L), reaching a maximum titer of 27.46 ± 0.10 mg/L at 36 h. Notably, L‐theanine accumulation declined markedly after 36 h (Figure S1), indicating that endogenous catabolic pathways may be degrading the product. In addition, the substantial accumulation of the glutamate and α‐KG intermediates suggests an imbalance between the available α‐KG pool from the Weimberg pathway and the flux directed toward L‐theanine synthesis (Figure 4c).

### 3.4 Blocking of L‐Theanine Degradation Pathways

**FIGURE 5:**
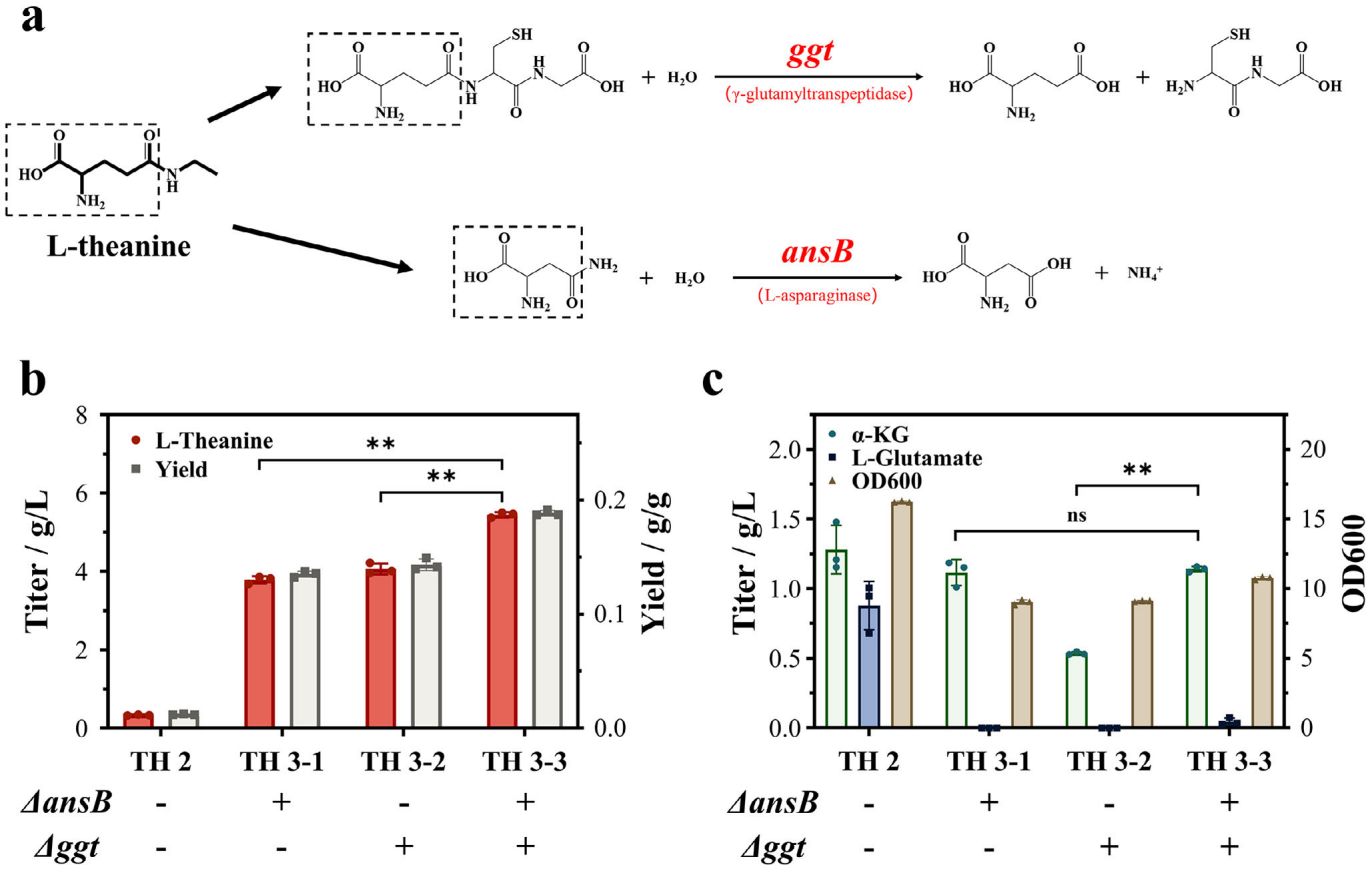
Blocking of L‐theanine degradation pathways. (a) The main decomposition pathway diagram of L‐theanine. Comparison of (b) L‐theanine production and yield, (c) glutamate, α‐KG accumulation, and biomass formation in different strains. *p* < 0.05 and ^**^ *p* < 0.01 indicate the significance level between the two engineered strains. Each experiment was repeated three times, and the data are represented as the means of three replicates, and bars represent the standard deviations.

Since the glutamyl group in L‐theanine is chemically similar to the amides in L‐glutamine or L‐asparagine, we hypothesized that the enzymes involved in glutamyl or asparaginyl transformation may also recognize L‐theanine as a substrate, thereby cleaving its amide bond. After excluding enzymes with high substrate specificity, we finally targeted two periplasmic enzymes, i.e., L‐asparaginase 2 (*ansB*) and γ‐glutamyltranspeptidase (*ggt*), both of which were previously reported to exhibit substrate promiscuity toward amide‐containing compounds (Figure 5a) [21].

To eliminate L‐theanine degradation, we sequentially inactivated these two genes in the parental strain TH 2 to generate single‐gene knockout strains TH 3‐1 (Δ*ansB*) and TH 3‐2 (Δ*ggt*), and a double knockout strain TH 3‐3 (Δ*ansB‐*Δ*ggt*). Although deletions of *ansB* and *ggt* slightly decreased cell growth, all knockout strains exhibited remarkably increased L‐theanine titers (Figure 5b), confirming that both genes contribute to the catabolism of L‐theanine. Among these strains, the double knockout strain TH 3‐3 achieved the highest L‐theanine titer, reaching 5.44 ± 0.07 g/L, which corresponds to a yield of 0.19 g/g xylose and represents a 16.2‐fold increase over that of strain TH 2 (Figure 5c). Furthermore, we exogenously supplemented L‐theanine in the culture of strain TH 3‐3, and no detectable L‐theanine degradation was observed (Figure S2), indicating that the catabolism of L‐theanine should be effectively eliminated.

### 3.5 Enhancing the Precursor Supply by GDH Selection and Cofactor Regulation

Glutamate dehydrogenase (GDH) catalyzes the NAD(P)H‐dependent reductive amination of α‐KG to glutamate, a rate‐limiting precursor for L‐theanine biosynthesis [23, 24]. Due to the Weimberg pathway metabolizing xylose generates excess NADH, we first overexpressed two NADH‐dependent GDHs with high enzymatic activity and good thermostability (GDH‐1 from *Amphibacillus xylanus* [25] and GDH‐2 from *Clostridium difficile* [26]) in the TH3‐3 strain, thereby obtaining the engineered strains TH4‐1 and TH4‐2. As a natural glutamate producer, *C. glutamicum* possesses high levels of GDH activity. Therefore, we also overexpressed two NADPH‐dependent GDHs derived from *E. coli* (GDH‐3) and *C. glutamicum* (GDH‐4), respectively, thereby obtaining the engineered strains TH4‐3 and TH4‐4.

**FIGURE 6:**
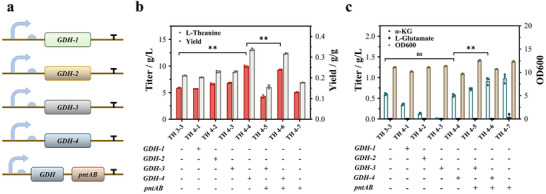
Enhancing the precursor supply by GDH selection and cofactor regulation. (a) GDH genes from different sources were introduced into engineered *E. coli*. Comparison of (b) L‐theanine production and yield, (c) glutamate, α‐KG accumulation, and biomass formation in differernt strains. ^*^ *p* < 0.05 and ^**^ *p* < 0.01 indicate the significance level between the two engineered strains. Each experiment was repeated three times, and the data are represented as the means of three replicates, and bars represent the standard deviations.

The experimental results of overexpressing the four GDHs are shown in Figure 6. The overexpression of GDH‐1 (strain TH 4‐1) did not improve L‐theanine production, whereas the expression of GDH‐2 (strain TH 4‐2) and GDH‐3 (strain TH 4‐3) showed a modest increase, with a final titer of 6.64 ± 0.16 g/L and 6.84 ± 0.12 g/L, respectively. Notably, strain TH 4‐4, which overexpresses GDH‐4, achieved the highest L‐theanine titer of 9.94 ± 0.18 g/L, a 68.8% increase relative to the control strain TH 3‐3, with a yield of 0.33 g/g (Figure 6b). Further analysis showed that no significant accumulation of α‐KG was observed in strain TH 4‐3 (Figure 6c), indicating the high catalytic efficiency of GDH‐4 compared to the other GDH variants.

A sufficient supply of NADPH is pivotal for achieving high‐level glutamate accumulation. While the Weimberg pathway converts xylose to α‐KG and generates 2 NADH per xylose molecule, the GDH reaction in strain TH 4‐4 requires 1 NADPH per cycle, leading to an inherent cofactor imbalance. In *E. coli*, the membrane‐bound transhydrogenase PntAB converts NADH into NADPH, and has proven effective in enhancing NADPH availability in amino acid overproduction [27, 28]. Accordingly, we overexpressed PntAB in the NADPH‐dependent backgrounds of strains TH 4‐3 (GDH‐3) and TH 4‐4 (GDH‐4), creating strains TH 4–5 and TH 4–6, respectively. However, no significant difference was observed between TH 4–6 and TH 4‐4, and overexpression of PntAB in the GDH‐3 background strain TH 4–5 even resulted in a 38% decrease in L‐theanine titer. This decline may be attributed either to the metabolic burden of additional protein expression, or to the extra ATP required for NADPH regeneration, because both the PntAB‐meditated NADPH regeneration and ATP synthesis rely on the proton motive force across the membrane [29, 30]. Notably, although the xylose‐to‐α‐KG conversion through the Weimberg pathway did not provide any NADPH, we hypothesized that the majority of NADPH required for GDH‐4 might be originated from the subsequent TCA cycle via NADPH‐dependent isocitrate dehydrogenase (IDH), which catalyzed oxidation of isocitrate to α‐KG.

### 3.6 Flux Balance Analysis Based on the Enzyme‐Constrained GEM

The TCA cycle is pivotal for cellular energy metabolism, yet its dual roles in central carbon metabolism and L‐theanine biosynthesis necessitate careful balancing. However, a critical metabolic trade‐off arises due to the following requirements: (i) the need to minimize α‐KG consumption, as it is a key precursor for L‐theanine synthesis, and (ii) the necessity of the TCA cycle for generating energy and reducing power, which are essential for cellular growth and L‐theanine production.

To better understand this metabolic trade‐off, we developed an enzyme‐constrained GEM derived from the widely used iML1515 model by incorporating L‐theanine biosynthesis and the Weimberg pathway for xylose metabolism. The resulting model includes 1882 metabolites and 2721 reactions, with enzyme kinetic parameters for key reactions integrated from DLKcat and other reputable enzyme databases. This approach ensures the model accurately reflects *E. coli* metabolism and allows for a more precise flux balance analysis, which can predict the optimal theoretical flux for L‐theanine synthesis and guide subsequent metabolic engineering strategies.

**FIGURE 7:**
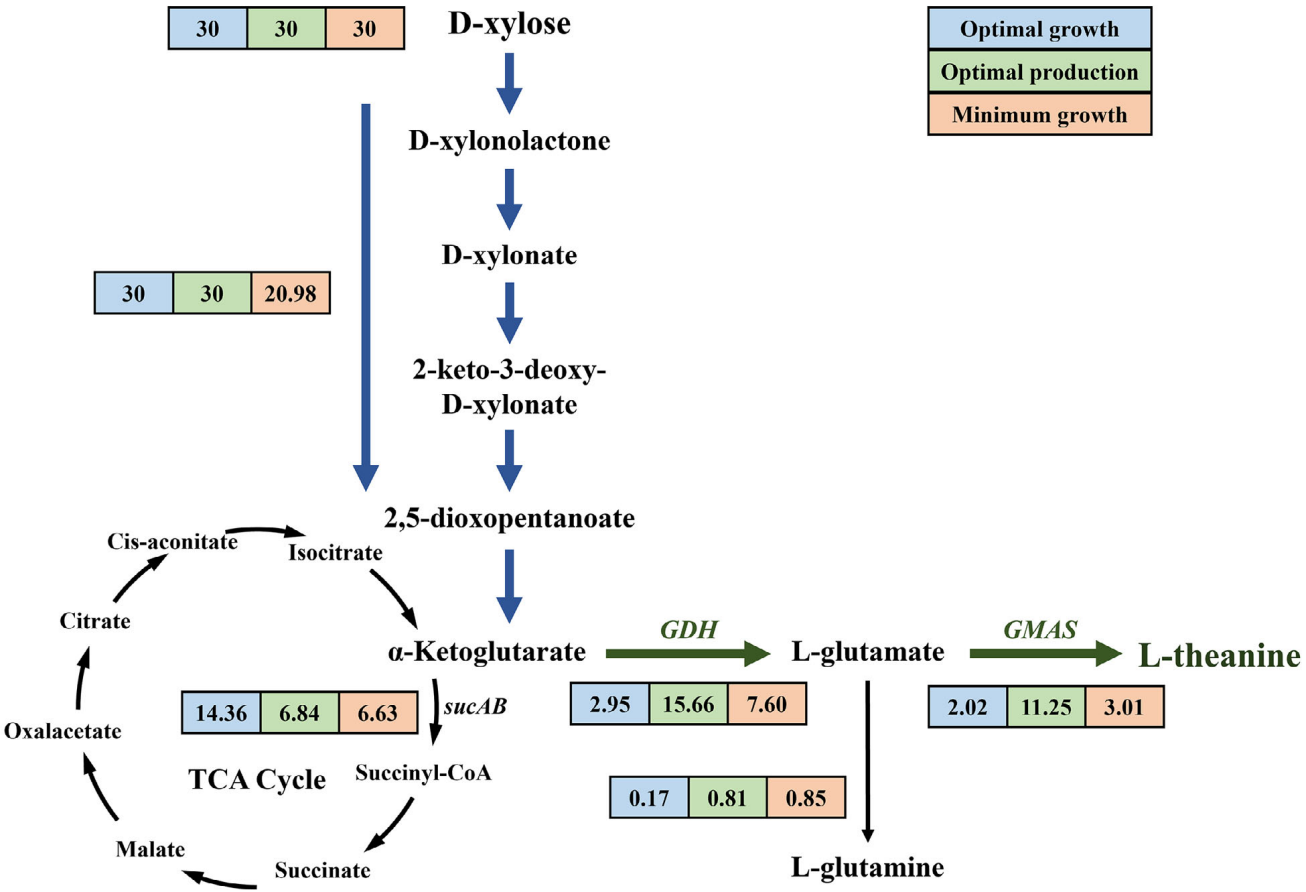
Flux balance analysis of the enzyme‐constrained metabolic network model for L‐theanine synthesis. The xylose uptake rate was set at 30 mmol/gDCW/h. Numbers in boxes represent metabolic flux values of reactions. Blue boxes indicate metabolic fluxes for maximum biomass formation. Green boxes represent metabolic fluxes for maximizing L‐theanine synthesis. Orange boxes denote metabolic fluxes for L‐theanine biosynthesis while maintaining the minimum growth requirement of the engineered strain.

We simulated three distinct scenarios to quantitatively assess metabolic trade‐offs: (i) maximum biomass production, (ii) maximum L‐theanine production under basal growth conditions, and (iii) minimal growth‐associated metabolism (Figure 7). Flux distribution analysis revealed a significant bifurcation between L‐theanine synthesis and the TCA cycle. Under maximum growth conditions, 47.9% of total carbon flux was allocated to central metabolism, while only 6.7% contributed to L‐theanine synthesis. Importantly, under basal growth conditions, a strategic downregulation of TCA flux effectively redirected carbon flow, increasing the L‐theanine flux from 6.7% to 37.5%. However, excessive suppression of central metabolism resulted in a substantial reduction in xylose metabolism flux to 20.98 mmol/gDCW/h, with 31.6% of the total flux being diverted to the TCA cycle and only 14.3% utilized for L‐theanine synthesis. Therefore, precisely downregulating the central carbon metabolism of the engineered strain can effectively increase L‐theanine production.

### 3.7 Fine‐Tuning the Carbon Flux of the Central Metabolic Pathway

#### 3.7.1 Fine‐Tuning the Carbon Flux of the Central Metabolic Pathway by Genetic Engineering

**FIGURE 8:**
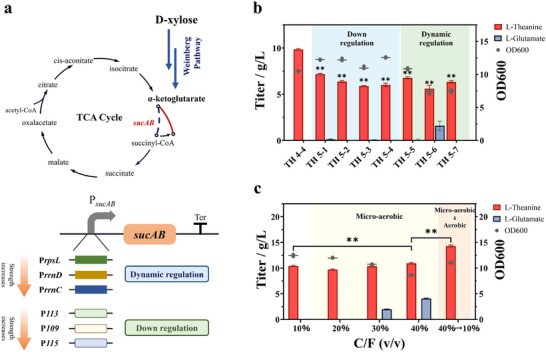
Fine regulation of central carbon metabolism. (a) Fine‐tuning the carbon flux of the central metabolic pathway by downregulating and dynamically regulating the *sucAB* genes. (b) The impact of downregulating or dynamically regulating the *sucAB* genes on L‐theanine yield and the biomass of engineered bacteria. (c) Improving L‐theanine production by modulating the central carbon metabolic flux through changes in dissolved oxygen levels. ^*^ *p* < 0.05 and ^**^ *p* < 0.01 indicate the significance level between the two engineered strains. Each experiment was repeated three times, and data are represented as the means of three replicates, and bars represent the standard deviations.

Computational predictions suggested that precise attenuation of central carbon metabolism in the engineered strain can be an effective strategy to enhance L‐theanine production. To this end, we implemented two genetic strategies to modulate the TCA cycle (Figure 8a): (i) statically transcriptional attenuation of *sucAB* (encoding α‐ketoglutarate dehydrogenase complex) via promoter or start codon modifications; and (ii) dynamically regulation of *sucAB* expression by growth‐phase‐dependent promoters (GPPs).

Initially, we replaced the native promoter of *sucAB* with three week constitutive promoters, i.e., *P*_J23115_ (iGEM Part: BBa_J23115), *P*_J23109_ (iGEM Part: BBa_J23109), and *P*_J23113_ (iGEM Part: BBa_J23113), which exhibit decreasing strengths, yielding strains TH 5‐1 to TH 5‐3. While final biomass remained comparable to the parent strain TH 4‐4, all modified strains exhibited prolonged lag phases (Figure S3). Critically, L‐theanine titers significantly decreased by 32–37%, potentially due to the early‐phase TCA suppression disrupting energy metabolism essential for biosynthesis. In addition, a parallel approach changing the start codon of *sucAB* from ATG to GTG in strain TH 5‐4 yielded similar results, suggesting that static attenuation strategies cannot effectively balance the flux between TCA cycle and L‐theanine biosynthesis.

To overcome the limitations of static regulation, we then designed a dynamic toggle switch by using the GPPs, which enables a real‐time modulation of carbon flux by controlling *sucAB* expression. Ideally, in this approach, *sucAB* expression remains high during the exponential growth phase to ensure sufficient TCA flux for robust cell growth, and is subsequently reduced upon entry into the stationary phase to divert more carbon flux toward L‐theanine production. Guided by this principle, we replaced the native promoter of *sucAB* with three different GPPs, i.e., PrrnC P1, PrrnD P1, and PrpsL, with decreasing expression strengths ranging from strong to weak, resulting in strains TH 5‐4 to TH 5–6 (Figure 8b).

Compared to the control strain TH 4‐4, the replacement with the PrrnC P1 promoter had minimal impact on growth, while replacing with PrrnD P1 and PrpsL resulted in reduced biomass accumulation. However, similar to the strains with static downregulation of the TCA cycle, the GPP‐modified strains did not exhibit increased L‐theanine production. This could be attributed to insufficient energy supply for L‐theanine biosynthesis due to the reduced TCA cycle activity as the strains entered the stationary phase.

#### 3.7.2 Fine‐Tuning the Carbon Flux of the Central Metabolic Pathway by Process Engineering

Given the limited success of genetic engineering in precisely regulating the carbon flux within the central metabolic pathway, we implemented a process engineering approach to achieve better flux control by modulating dissolved oxygen (DO) via culture volume adjustments. Specifically, we varied the ratio of culture medium volume to total flask volume (C/F: 10%‐40%, v/v), which altered dissolved oxygen levels and created a gradient of microaerobic conditions. This gradient indirectly regulated the flux to the TCA cycle, offering a straightforward approach that allows for the creation of diverse OD gradients, thus enabling more precise and targeted control over carbon flux.

As shown in Figure 8c, increasing the C/F ratio from 10% to 40% resulted in a slight increase in L‐theanine production, with the highest titer reaching 11.2 g/L at a C/F ratio of 40%. However, a significant accumulation of L‐glutamate was observed with C/F ranging from 30% to 40%. This accumulation should be attributed to the insufficient ATP supply for the enzyme GMAS‐A, which catalyzes the conversion of L‐glutamate to L‐theanine at the expense of ATP. This observation aligns with results from experiments where the *sucAB* gene expression was attenuated.

To further resolve the ATP supply shortage during the L‐theanine production, we implemented a two‐stage microaerobic‐aerobic cultivation strategy (Figure 8c). During the growth phase, DO levels were reduced to prevent excessive consumption of central carbon metabolism. In the production phase, oxygen supply was increased to ensure adequate energy availability for L‐theanine synthesis. This two‐stage strategy effectively enhanced L‐theanine production, achieving a titer of 14.31 ± 0.21 g/L with a yield of 0.48 g/g xylose, which is 1.44 times higher than that under aerobic cultivation conditions.

### 3.8 Fed‐Batch Production of L‐Theanine in a 5 L Bioreactor

To evaluate the scale‐up potential of L‐theanine production, a fed‐batch fermentation was conducted using strain TH4‐4 in a 5 L bioreactor. Through systematic optimization, which included staged dissolved oxygen control along with precise feeding strategies for xylose, ethylamine hydrochloride, and other key additives (Figure S4), the titer and yield of theanine were significantly improved. During the fermentation, cell growth entered the stationary phase within 20 h, and the OD600 remained around 50 until the process ended.

**FIGURE 9:**
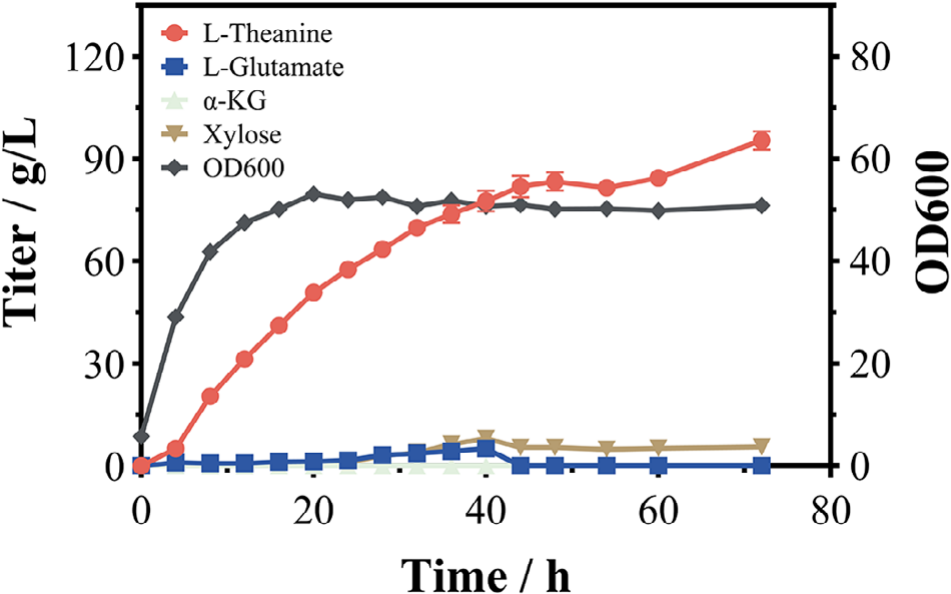
Batch‐fed‐batch production of L‐theanine in a 5 L bioreactor.

As shown in Figure 9, L‐theanine was accumulated rapidly after 4 h, reaching a peak concentration of 95.42 ± 2.65 g/L at 72 h. The corresponding yield and productivity were 0.55 g/g xylose and 1.33 g/L/h, respectively. In addition, concentrations of by‐products such as glutamate and other organic acids stayed at low levels throughout the fermentation, indicating that L‐theanine could be efficiently recovered from the fermentation broth, potentially lowering downstream separation and purification costs. Further optimization of carbon flux distribution between the L‐theanine synthesis pathway and the TCA cycle is expected to enhance xylose utilization efficiency. By integrating multi‐omics approaches such as metabolomics and fluxomics, it will be possible to systematically analyze intracellular metabolic networks, identify key metabolic engineering bottlenecks, and achieve continuous improvement in theanine production performance.

## 4 Discussion

**FIGURE 10:**
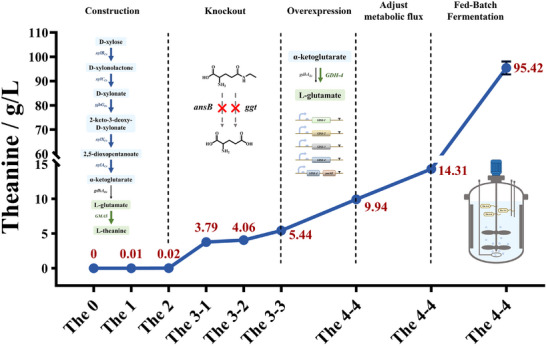
Regulation strategies of engineered *Escherichia coli* for *de novo* synthesis of L‐theanine.

Microbial production of L‐theanine from D‐xylose is fundamentally constrained by the carbon inefficiency of central metabolism. For instance, the native pentose phosphate pathway (PPP) dissipates over 17% of carbon as CO_2_, thereby limiting the maximum theoretical yield. To address this challenge, we engineered a synthetic microbial platform in *E. coli* that integrates the carbon‐conserving Weimberg pathway for xylose assimilation with model‐guided optimization (Figure 10). This integrated approach not only achieves the highest reported titer of 95.42 g/L and yield of 0.55 g/g, but also demonstrates a scalable and economically attractive route for producing TCA cycle‐derived chemicals. As summarized in Table 2, our work significantly outperforms previous microbial processes, including those utilizing preferred substrates like glucose, thereby setting a new benchmark in the field.

**TABLE 2:** Comparison of L‐theanine synthesis by biological fermentation.

Strain	Substrate	Titer (g/L)	Yield (g/g)	Key Enzyme	References
*C. glutamicum*	Glucose	42.00	0.19	GMAS_Mm_	[18]
*C. glutamicum*	Glucose	44.12	0.55	GMAS_Pa_	[19]
*P. putida*	Xylose	4.00	0.02	GMAS_Me_	[21]
*S. cerevisiae*	Glucose	48.19	—	CsTSI, CsAlaDC	[31]
*E. coli*	Glucose	70.60	0.42	GMAS_Pa_	[20]
*E. coli*	Glucose	16.10	0.13	GMAS_Ps_, CsAlaDC	[22]
*E. coli*	Xylose	95.42	0.55	GMAS_Pa_	This study

We first established a platform strain capable of efficiently utilizing xylose to synthesize the intermediate α‐ketoglutarate (α‐KG) by disrupting the PPP pathway through the deletion of the native *xylA* gene and replacing the rate‐limiting gene *xylD* with *yjhG* gene from *E. coli* MG1655. After optimizing the key enzymes involved in the pathway, including the GMAS enzyme, we observed a significant increase in L‐theanine production. The overexpression of GMAS‐A from *P. aminovorans* resulted in a higher L‐theanine yield, reaching 5.44 ± 0.07 g/L after deleting the *ansB* and *ggt* genes, which eliminated the degradation of L‐theanine (Figure 5b). This marks a 16.2‐fold increase in L‐theanine titer compared to the control strain, demonstrating the importance of gene knockout strategies in optimizing product yields.

Further optimization focused on enhancing the supply of α‐KG and L‐glutamate, which are essential precursors for L‐theanine synthesis. The overexpression of various GDH enzymes, particularly the NADPH‐dependent GDH‐4, was successful in increasing the L‐theanine yield to 9.94 ± 0.18 g/L, a 68.8% improvement over the control strain (Figure 6). This result underscores the critical role of GDH in channeling α‐KG into the L‐theanine biosynthesis pathway. Moreover, the cofactor balance between NADH and NADPH was optimized by overexpressing PntAB, a membrane‐bound transhydrogenase, which was intended to enhance NADPH availability. However, no significant improvement in L‐theanine production was observed when *pntAB* was overexpressed in the GDH backgrounds, suggesting that NADPH generation was not the limiting factor in L‐theanine synthesis, and other factors, such as ATP supply, played a more prominent role.

One of the most significant challenges in optimizing the L‐theanine production pathway was balancing the flux between the TCA cycle and L‐theanine biosynthesis. Flux balance analysis (FBA) revealed a competitive relationship between these two processes, with the TCA cycle consuming a substantial portion of the available carbon flux, thereby limiting the amount directed toward L‐theanine production (Figure 7). To address this issue, we employed various strategies to modulate the TCA cycle, including the dynamic regulation of *sucAB* expression via growth‐phase‐dependent promoters and controlling dissolved oxygen levels in the culture medium. While static downregulation of the TCA cycle did not yield significant improvements in L‐theanine production, the two‐stage microaerobic‐aerobic strategy successfully addressed ATP limitations by providing sufficient oxygen during the production phase, resulting in a significant increase in L‐theanine yield (Figure 8c). This strategy led to a titer of 14.31 ± 0.21 g/L, a productivity of 0.48 g/g xylose, and a 1.44‐fold increase in yield compared to aerobic cultivation.

The scalability of the process was further demonstrated in a 5 L fed‐batch fermentation system, where the engineered strain TH 4‐4 achieved a final L‐theanine titer of 95.42 ± 2.65 g/L at 72 h, with a yield of 0.55 g/g and productivity of 1.33 g/L/h (Figure 9). The ethylamine tolerance assay showed that the concentration of ethylamine during fermentation was far below its toxicity threshold and did not exert any substantial adverse effect on cell growth (Figure S5). Notably, the low levels of by‐products such as L‐glutamate and other organic acids during fermentation suggested that L‐theanine could be easily recovered from the broth, which could significantly reduce downstream separation and purification costs. This result highlights the practical potential of our engineered platform for large‐scale production of L‐theanine.

In conclusion, our study provides a robust platform for L‐theanine production from xylose in *E. coli*, combining systematic metabolic engineering and GEM‐guided optimization to achieve high yields and productivity. The non‐phosphorylative pathway established here not only provides a promising route for L‐theanine synthesis but also offers broader potential for the production of other valuable metabolites derived from the TCA cycle. The successful application of this platform in large‐scale fermentation demonstrates its commercial viability, and future efforts to further optimize the strain and fermentation conditions will likely lead to even higher production rates and lower operational costs. Additionally, future efforts could focus on the co‐utilization of more cost‐effective substrates, such as glucose or acetate, which would further lower production costs and enhance the economic feasibility of the process.

## Author Contributions

Haolin Han: Writing – review & editing, Writing – original draft, Conceptualization, Data curation, Formal analysis, Investigation, Methodology, Project administration, Supervision, Visualization. Boyuan Xue: Writing – original draft, Conceptualization, Data curation, Methodology, Software. Guangqi Shan: Writing – original draft, Data curation, Formal analysis, Methodology. Meng Meng: Writing – original draft, Data curation, Formal analysis, Methodology. Shaojie Wang: Writing – review & editing, Conceptualization, Data curation, Formal analysis, Funding acquisition, Methodology, Project administration. Haijia Su: Writing – review & editing, Conceptualization, Data curation, Formal analysis, Funding acquisition, Methodology, Project administration.

## Conflicts of Interest

The authors declare no conflicts of interest.

## Supporting information




**Supporting File**: advs73770‐sup‐0001‐SuppMat.docx.

## Data Availability

The data that support the findings of this study are available from the corresponding author upon reasonable request.
