# Supplementary material for: Systematic Metabolic Engineering and Model‐Guided Optimization for High‐Level Production of L‐Theanine from Xylose in Escherichia coli
Source: Adv Sci (Weinh). 2026 Jan 21;13(17):e21440. doi: 10.1002/advs.202521440 (PMC13042605; doi:10.1002/advs.202521440)
Supplement: Supplementary file 1 — Supporting File: advs73770‐sup‐0001‐SuppMat.docx. [file ADVS-13-e21440-s001.docx]

**Supplementary materials**

**Systematic Metabolic Engineering and Model-Guided Optimization for High-Level Production of L-Theanine from Xylose in *Escherichia coli***

Haolin Han, Boyuan Xue, Guangqi Shan, Meng Meng, Shaojie Wang*, Haijia Su*

State Key Laboratory of Green Biomanufacturing, National Energy R&D Center for Biorefinery, Beijing Key Laboratory of Green Chemicals Biomanufacturing, Beijing Synthetic Bio-manufacturing Technology Innovation Center, Beijing University of Chemical Technology, Beijing, 100029, People’s Republic of China

*Corresponding author: suhj@mail.buct.edu.cn, wangshaojie@buct.edu.cn

**Figure S1 L-theanine production of TH 2 strain.**

**Figure S2. Analysis of L-theanine degradation in engineered strain TH 4 with exogenous L-theanine supplementation.**


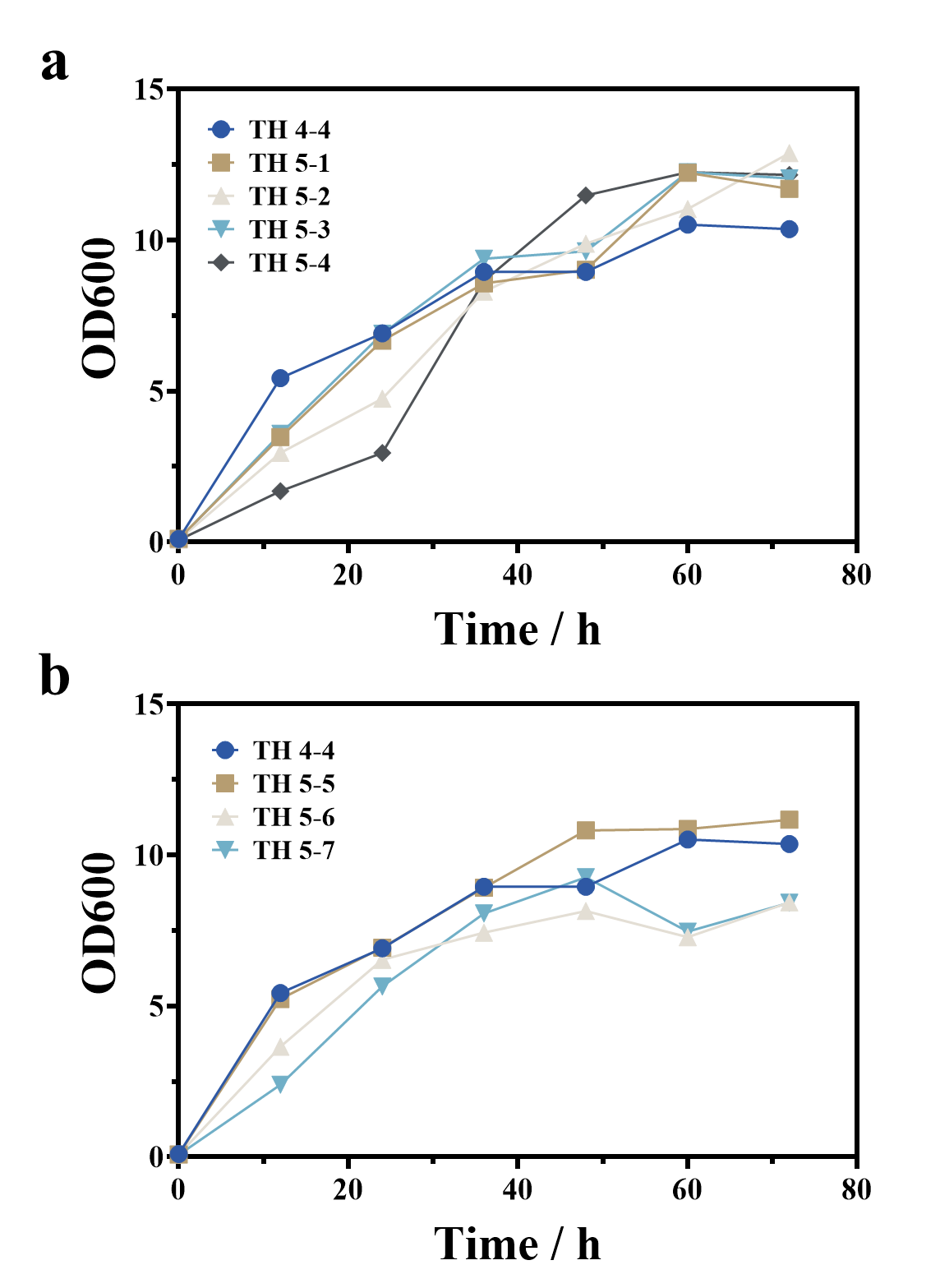


**Figure S3. Effect of *sucAB* gene regulation on bacterial growth.** (a) Comparison of growth curves of strains with down-regulated *sucAB* gene. (b) Comparison of growth curves of strains with dynamic regulation of *sucAB* gene.


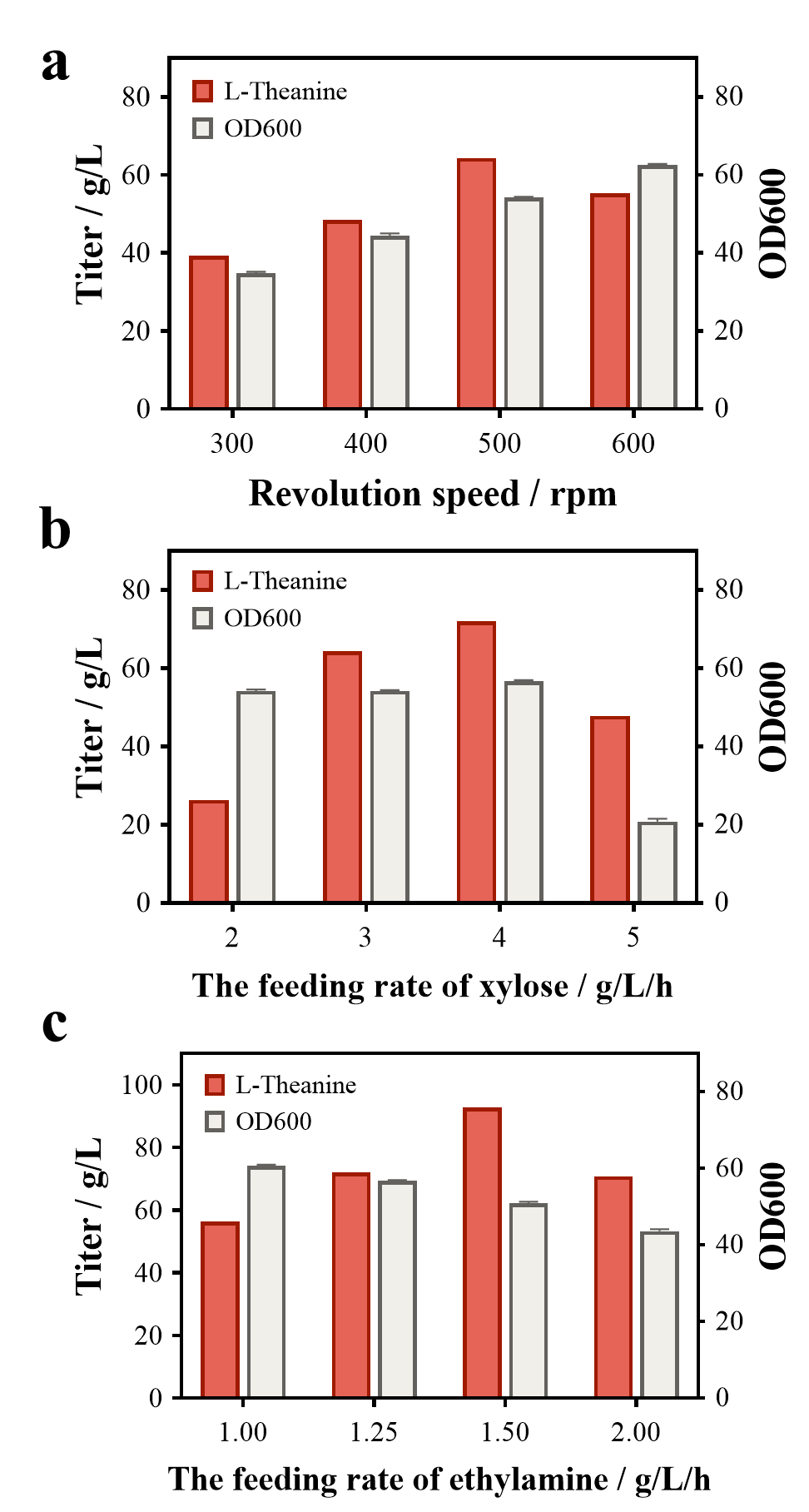


**Figure S4. Optimization of culture conditions for fed-batch fermentation.** (a) Effect of dissolved oxygen conditions on L-theanine production. (b) Effect of xylose feeding rate on theanine production. (c) Effect of feeding rate of ethylamine on theanine production.

**Figure S5. Ethylamine tolerance test of the engineered strain.**

**Table S1 Strains and plasmids used in this study.**

| Strains / plasmids | Description | Ref. |
| --- | --- | --- |
| **Strains** |  |  |
| *E. coli* DH5α | Host for cloning | Lab stock |
| *E. coli* BL21(DE3) | Wild type, starting strain | Lab stock |
| W 0 | BL21, △*xylA* | This study |
| W 1 | W 0 harboring plasmid pW 1 | This study |
| W 2 | W 0 harboring plasmid pW 2 | This study |
| TH 1 | W 2 harboring plasmid pTH 1 | This study |
| TH 2 | W 2 harboring plasmid pTH 2 | This study |
| TH 3-1 | TH 2, △*ansB* | This study |
| TH 3-2 | TH 2, △*ggt* | This study |
| TH 3-3 | TH 2, △*ansB*, △*ggt* | This study |
| TH 4 | W 2, △*ansB*, △*ggt* | This study |
| TH 4-1 | TH 4 harboring plasmid pTH 3-1 | This study |
| TH 4-2 | TH 4 harboring plasmid pTH 3-2 | This study |
| TH 4-3 | TH 4 harboring plasmid pTH 3-3 | This study |
| TH 4-4 | TH 4 harboring plasmid pTH 3-4 | This study |
| TH 4-5 | TH 4 harboring plasmid pTH 3-5 | This study |
| TH 4-6 | TH 4 harboring plasmid pTH 3-6 | This study |
| TH 4-7 | TH 4 harboring plasmid pTH 3-7 | This study |
| TH 5-1 | TH4-4, replacing native promoter of *sucAB* with P_BBa_J23109_ | This study |
| TH 5-2 | TH4-4, replacing native promoter of *sucAB* with P_BBa_J23115_ | This study |
| TH 5-3 | TH4-4, replacing native promoter of *sucAB* with P_BBa_J23113_ | This study |
| TH 5-4 | TH4-4, replacing the start codon of *sucAB* from ATG to GTG | This study |
| TH 5-5 | TH4-4, replacing native promoter of *sucAB* with P_rrnC_ | This study |
| TH 5-6 | TH4-4, replacing native promoter of *sucAB* with P_rrnD_ | This study |
| TH 5-7 | TH4-4, replacing native promoter of *sucAB* with P_rpsL_ | This study |
| **Plasmids** |  |  |
| pEcCas9 | Spe, expression of Cas9 and λRed recombinase | [1] |
| pEcgRNA | Kan, gRNA expression vector | [1] |
| pACYC-Duet | Expression plasmid, lacI, T7 promoter, Cm | Lab stock |
| pET-Duet | Expression plasmid, lacI, T7 promoter, Amp | Lab stock |
| pEc-Δ*xylA* | Derived from pEcgRNA, target *xylA* in *E. coli* BL21(DE3) | This study |
| pEc-Δ*ansB* | Derived from pEcgRNA, target *ansB* in *E. coli* BL21(DE3) | This study |
| pEc-Δ*ggt* | Derived from pEcgRNA, target *ggt* in *E. coli* BL21(DE3) | This study |
| pW 1 | pACYC-*xylBCDAX_Cc_* | This study |
| pW 2 | pACYC-*xylBCAX_Cc_*-*yjhG_Ec_* | This study |
| pTH 1 | pET-*GMAS-S* | This study |
| pTH 2 | pET-*GMAS-A* | This study |
| pTH 3-1 | pET-*GMAS-A*-*GDH-1* | This study |
| pTH 3-2 | pET-*GMAS-A*-*GDH-2* | This study |
| pTH 3-3 | pET-*GMAS-A*-*GDH-3* | This study |
| pTH 3-4 | pET-*GMAS-A*-*GDH-4* | This study |
| pTH 3-5 | pET-*GMAS-A*-*GDH-3*-*pntAB* | This study |
| pTH 3-6 | pET-*GMAS-A*-*GDH-4*-*pntAB* | This study |
| pTH 3-7 | pET-*GMAS-A*-*pntAB* | This study |

Reference

[1] Li, Q. I., Sun, B., Chen, J., Zhang, Y., Jiang, Y. U., & Yang, S. (2021). A modified pCas/pTargetF system for CRISPR-Cas9-assisted genome editing in *Escherichia coli*. *Acta Biochimica et Biophysica Sinica*, *53*(5), 620-627.

**Table S2 Primers used in this study.**

| Primer | Sequence (5´-3´) |
| --- | --- |
| pEcgRNA-F | aagcttagatctattaccctgttatccctac |
| pEcgRNA-R | atgtcaggctcccttatacacagc |
| gRNA-R | gaattcaaaaaaagcaccgac |
| xylA-N20-F | tgtataagggagcctgacatCAGGAAGCCATAGACCGTCGgttttagagctagaaatagc |
| xylA-Up-F | tcggtgctttttttgaattcttgagcaactgaaagggagtgcc |
| xylA-Up-R | ggctatttcaggttccccacagcagcttc |
| xylA-Down-F | gtggggaacctgaaatagccaccgccattgc |
| xylA-Down-R | agggtaatagatctaagcttcggactgcacagttagccg |
| ansB-N20-F | tgtataagggagcctgacatTCCATTCAACCTGTATAACGgttttagagctagaaatagc |
| ansB-Up-F | tcggtgctttttttgaattcttagtactgattgaagatctgctgg |
| ansB-Up-R | gcttacttccgccgaaagtcggcattgtttataac |
| ansB-Down-F | gactttcggcggaagtaagcggtttcttc |
| ansB-Down-R | agggtaatagatctaagcttatggagtttttcaaaaagacg |
| ggt-N20-F | tgtataagggagcctgacatATGCCACCGCCATCCTCCGGgttttagagctagaaatagc |
| ggt-up-F | tcggtgctttttttgaattcttagtaccccgccgttaaatcatcc |
| ggt-up-R | tcgtgcagccggcggtgacctatacgctg |
| ggt-down-F | ggtcaccgccggctgcacgactttgttc |
| ggt-down-R | agggtaatagatctaagcttatgataaaaccgacgtttttacg |
| pACYC-F | ggatccgaattcgagctcg |
| pACYC-R | ggtatatctccttattaaagttaaacaaaattatttctaca |
| xylB-F | ctttaataaggagatataccatgagtagcgccatttacccgag |
| xylB-R | ccttagatccttagcgccagcccgcatc |
| xylC-F | ctggcgctaaggatctaaggagatatacatatgaccgctcaggtaactt |
| xylC-R | ccttagatccttaaaccaggcgcacttcg |
| xylD-F | cctggtttaaggatctaaggagatatacatatgagcaaccgtaccccg |
| xylD-R | gagctcgaattcggatccttagtgattatggcgtggcagtt |
| yjhG-F | ggatctaaggagatatacatatgtctgttcgcaatatttttg |
| yjhG-R | ccgagctcgaattcggatcctcagtttttattcataaaatcgcg |
| xylX-F | agatatacatatgggcgtgagcgagtttc |
| xylX-R | atgtatatctccttagatccttacagcagaccgcgaccggcc |
| xylA-F | ggatctaaggagatatacatatgaccgatacgctgcgc |
| xylA-R | agcggtggcagcagcctaggttagctccagctgtagctgg |
| pET-F | ggatccgaattcgagctcg |
| pET-R | ggtatatctccttcttaaagttaaacaaaattatttctagaggg |
| GMAS-A-F | ctttaagaaggagatataccatgaccgatctggccgaatttg |
| GMAS-A-R | ctgtcatgatatgtatatctccttagatccttaaatatctaaagtgtgatcgcgttcc |
| GDH-4-F | ggatctaaggagatatacatatcatgacagttgatgagcaggtctc |
| GDH-4-R | ccgagctcgaattcggatccttagatgacgccctgtgccag |
| GDH-1-F | tataagaaggagatatacatatgacccaagataactataacgcgtatc |
| GDH-1-R | agcggtggcagcagcctaggttaaatccagccgcgcagtttc |
| GDH-2-F | tataagaaggagatatacatatgagcggcaaagatgtgaacg |
| GDH-2-R | agcggtggcagcagcctaggttaataccagccgcgcagtttc |
| GDH-3-F | tataagaaggagatatacatatggatcagacatattctctgg |
| GDH-3-R | agcggtggcagcagcctaggttaaatcacaccctgcgc |
| pntAB-F | tataagaaggagatatacatatgcgaattggcataccaagagaac |
| pntAB-R | agcggtggcagcagcctaggttacagagctttcaggattgcatccac |
